# Solvent Model Benchmark for Molecular Dynamics of Glycosaminoglycans

**DOI:** 10.1021/acs.jcim.2c01472

**Published:** 2023-03-29

**Authors:** Mateusz Marcisz, Sergey A. Samsonov

**Affiliations:** † Faculty of Chemistry, University of Gdańsk, ul. Wita Stwosza 63, 80-308 Gdańsk, Poland; ‡ Intercollegiate Faculty of Biotechnology of UG and MUG, ul. Abrahama 58, 80-307 Gdańsk, Poland

## Abstract

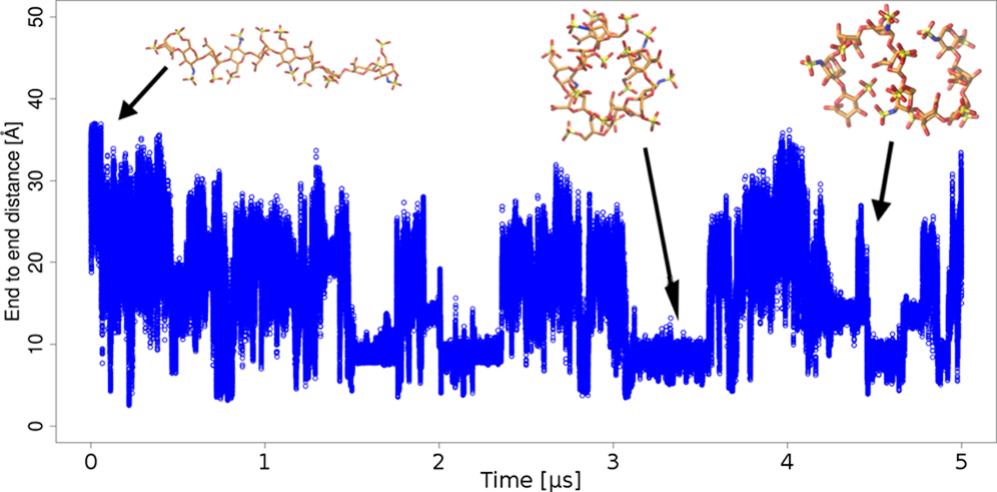

In computational studies of glycosaminoglycans (GAGs), a group of anionic, periodic linear polysaccharides, so far there has been very little discussion about the role of solvent models in the molecular dynamics simulations of these molecules. Predominantly, the TIP3P water model is commonly used as one of the most popular explicit water models in general. However, there are numerous alternative explicit and implicit water models that are neglected in the computational research of GAGs. Since solvent-mediated interactions are particularly important for GAG dynamic and structural properties, it would be of great interest for the GAG community to establish the solvent model that is suited the best in terms of the quality of theoretically obtained GAG parameters and, at the same time, would be reasonably demanding in terms of computational resources required. In this study, heparin (HP) was simulated using five implicit and six explicit solvent models with the aim to find out how different solvent models influence HP’s molecular descriptors in the molecular dynamics simulations. Here, we initiate the search for the most appropriate solvent representation for GAG systems and we hope to encourage other groups to contribute to this highly relevant subject.

## Introduction

Glycosaminoglycans (GAGs) are a group of anionic polysaccharides that are built of repeating disaccharide units.^1^ They are long periodic linear sulfated and highly charged polymers that exhibit different sulfation patterns, which may alter their binding and functional properties as well as conformational characteristics.^2,3^ There are six groups of mammalian GAGs: heparan sulfate (HS), heparin (HP), hyaluronic acid (HA), chondroitin sulfate (CS), dermatan sulfate (DS), and keratan sulfate (KS). Depending on the arrangement of the disaccharide units in those molecules, they may display 408 variants,^1^ of which 202 can be found in mammals.^4,5^ Their diversity contributes to the multifunctional role they play in the extracellular matrix of the cell affecting different types of processes such as cell signaling,^6^ cardiovascular diseases,^7^ cell maturation,^8^ tissue regeneration,^9,10^ proliferation,^11^ inflammatory response,^12^ infection,^6,13^ and diseases such as Alzheimer’s and Parkinson’s diseases^14^ or cancer development.^15,16^ It has been shown recently that GAGs also play a crucial role in the Sars-Cov-2 viral infection mechanism.^17−20^ All of these mentioned processes are affected directly by GAG intermolecular interactions with proteins: chemokines,^21−23^ growth factors,^24−26^ morphogenes,^27^ membrane receptors integrins,^28^ and lipoproteins.^29^ The majority of these interactions are driven by electrostatics and are nonspecific,^30,31^ while some of them, in contrast, can be highly specific^32^ or selective.^33^ The properties of the GAGs make it extremely challenging to study them using computational tools.^34^ Additionally, despite some of the recent advances in the field of GAG docking,^35−40^ it still lags behind the abundance of approaches designed for small drug molecules. Although at the moment, there are even several web servers that could be used for GAG docking,^41−43^ they do not perform as well as some conventional docking software (e.g., DOCK and Autodock3) for the highly heterogeneous data set of protein–GAG complexes.^44^

Most of the molecular dynamics (MD)-related studies on GAGs are conducted using the TIP3P water model as it is widely accepted in the GAG field and proven to be working well in the protein–GAG systems,^36,45−48^ as well as in MD studies of diverse biomolecular systems in general. The basic reason for the wide use of the TIP3P and other three-site water models is their low computational cost compared to four- and five-site solvent models. Implicit water models are used less frequently, especially nowadays when researchers have easier access to high-performance computing facilities than before. However, due to the lower computational costs of implicit solvent, this type of water model is still utilized when computational resources and time are limited or the size of the studied system is particularly big. Regarding the impact of the solvent type in computational science, some studies show that explicit solvent can improve the general quality of docking and MD simulations,^36,49−53^ while there is an evidence that GB models do not reproduce secondary structures of *de novo* designed peptides.^54^ To this day, there has been no exhaustive comparative study conducted on water models that supposes to answer the question which one has advantages to be used in systems involving GAG molecules. Previously, to our knowledge, there was one attempt to compare HP properties in the MD simulations with SPC and SPC/E water models.^55^ However, this study was conducted in GROMACS as opposed to AMBER used in this work with GLYCAM06.^56^ Additionally, the simulations were only 3 ns long, which is not enough to observe major conformational changes and which is far shorter than the state-of-the-art MD simulation length established standards nowadays. While there has been a general opinion based on common sense and studies not involving GAGs^57,58^ that it would be beneficial to use a more advanced water model than three-site TIP3P, e.g., TIP4P or TIP5P, there are no data on the use of different water models and thus no evidence which and if other model should be commonly used. This discussion is of immense importance due to the fact that in the available experimental structures half of the protein–GAG residue contacts are mediated by water and that there are about 10 times more water molecules in the protein–GAG interfaces than in the protein–protein interfaces.^59^ Thus, it is necessary to accurately model water-mediated interactions in protein–GAG complexes, especially taking into account the importance of the electrostatic interactions involved there.^49^ In fact, it was shown that interactions between proteins and GAGs can be stabilized by solvent molecules functioning as structured water in those complexes.^33,60−63^ It was also reported that the effect of the dynamical behavior of the solvent surrounding GAGs on the conformation of the saccharides is of great relevance.^64−66^ Taking all of this information into account, it would be beneficial for the computational GAG community to study the influence of the water models on the GAG behavior in the MD simulation.

In this work, we aim to evaluate the properties of the HP in different implicit and explicit water models to find out which of them are best suited for the MD simulation of GAG molecules. For this, 5 μs MD simulations were performed involving dp10 (dp stays for the degree of polymerization) HP molecule with the following water models: implicit IGB = 1,^67^ 2,^68^ 5,^69^ 7,^70^ and 8,^71^ and explicit TIP3P,^72,73^ SPC/E,^74^ TIP4P,^72^ TIP4PEw,^75^ OPC,^57^ and TIP5P.^76^ Additionally, five 200 ns MD simulations were performed for each of the setups as a consistency check for the convergence of the analyzed parameters. Using the obtained trajectories, HP properties such as end-to-end distance (EED), volume, radius of gyration, ring puckering, intramolecular hydrogen bonds, and dihedral angles were analyzed.

## Materials and Methods

### Heparin Structure and Parameterization for the MD Simulations

The initial structure of the HP dp10 used in this study was obtained from the Protein Data Bank,^77^1HPN structure.^78^ Literature data for the sulfate group charges^79^ and GLYCAM06^56^ force field parameters were used. ^1^C_4_ conformation for the IdoA2S ring was chosen as it was shown to be the essentially dominant conformation in the microsecond scale simulations performed by Sattelle *et al.* as it is energetically more favorable than the ^2^S_O_ conformation.^80^ Additionally, another study conducted by Bojarski *et al*. claims that the IdoA2S ^1^C_4_ pucker conformation reproduces a more probable and extended HP structure than the ^2^S_O_ conformation.^81^ The same study indicates that ^1^C_4_ is the preferable conformation and that when starting from the ^2^S_O_ conformation, it changes to the ^1^C_4_ one during the long MD simulation. Moreover, NMR studies also find that IdoA2S ^1^C_4_ conformation in HP oligosaccharides is dominant.^82,83^ Additionally, it was reported that HP’s IdoA2S ring conformation has little impact on other properties (such as radius of gyration and end-to-end distance) of the HP molecule.^84^

### Water Models

All of the parameters for the water models used in this work are taken from Amber16 and recommended mbondi were used for each particular model. The models used were implicit IGB = 1, 2, 5, 7, 8 and explicit TIP3P, SPC/E, TIP4P, TIP4PEw, OPC, and TIP5P. Graphical representation and the details of the solvent models used in this study are presented in Figure S1.

### MD Simulations

MD simulations have been carried out in the AMBER package.^85^ HP decasaccharide was solvated in an octahedral periodic box with a minimum distance between solute and box edge of 8.0 Å and neutralized with counterions (Na^+^) in the case of the explicit solvent simulations. No “saltcon” option was used in the implicit solvent simulations. Two energy minimization steps were carried out (first 1.5 × 10^3^ steepest descent cycles and 10^3^ conjugate gradient cycles with harmonic force restraints on solute atoms, followed by 6 × 10^3^ steepest descent cycles and 3 × 10^3^ conjugate gradient cycles without restraints) for the explicit solvent simulations, while only the second minimization step was performed for the implicit solvent simulations. Subsequently, the system was heated up to 300 K for 10 ps with harmonic force restraints of 100 kcal mol^–1^ Å^–2^ on solute atoms, and equilibration for 50 ps at 300 K and 10^5^ Pa in the isothermal isobaric ensemble (NPT) for the explicit solvent simulation. A productive MD run was also carried out in an NPT ensemble in the explicit solvent simulations. The SHAKE algorithm, 2 fs time integration step, 8 Å cutoff for nonbonded interactions, and the particle mesh Ewald method were used.

### Molecular Mechanics Generalized Born Surface Area (MM/GBSA) Analysis

Molecular mechanics generalized Born surface area (MM/GBSA) model IGB = 2 from AMBER16 was used for free energy calculations on the trajectories obtained from MD simulations in the case of the explicit water models. For the implicit water models, IGB values were chosen according to the model used in MD (e.g., IGB = 1 for IGB = 1 water model).^88^

### Heparin’s Properties

The radius of gyration, end-to-end distance (EED), hydrogen bonds, ring puckers, and dihedral angles were obtained using cpptraj scripts from the AMBER suite. The volume of the HP molecule (similar to the work of Nagarajan *et al*.^89^) was calculated using an in-house script. For this, we extracted the molecule’s coordinates from each frame of the trajectory, and afterward, the volume was assessed using mvee_REX (minimum-volume enclosing ellipsoid) from the OptimalDesign library in the R.^90^

The dihedral angles for glycosidic linkage analysis were defined as O5_*n*+ 1_–C1_*n*+ 1_–O4*_n_*–C4*_n_* and C1_*n*+ 1_–O4*_n_*–C4*_n_*–C3*_n_*, where *n* stands for the sequential number of a sugar monomeric unit. The dihedral angles for the ring puckering were defined as C1*_n_*–C2*_n_*–C3*_n_*–C4*_n_* and C1*_n_*–O5*_n_*–C5*_n_*–C4*_n_*. AMBER’s atom and residue numbering and nomenclature are used for the glycosidic linkage and ring puckering definitions. Visual representation of the dihedral angles (glycosidic linkages and puckering) is shown in Figure S2.

## Results

In order to observe changes in HP properties depending on the used water model, we performed 5 μs MD simulations for all of the investigated models. Then, additionally, we supplemented the data with shorter (five 200 ns for each model) simulations in order to check the consistency of the results and to assure the reproducibility of those simulations. Those relatively short simulations should allow us to compare HP conformational changes between different timescales.

HP behaved drastically differently in implicit and explicit water models. Naturally, there was a significant contrast between particular implicit and explicit water models, but those changes were less significant than when comparing GB implicit models with the explicit models (Tables 1, S1, and S2). The obtained data were compared to the experimental findings of Mulloy *et al*.,^78^ Khan *et al*.,^86^ and Pavlov *et al*.^87^

**Table 1:** HP Descriptors Obtained from 5 μs MD Simulations

	PDB 1HPN	IGB = 1	IGB = 2	IGB = 5	IGB = 7	IGB = 8	TIP3P	SPC/E	TIP4P	TIP4PEw	OPC	TIP5P
dist^a^ (Å)	41.0	40.4 ± 2.9	41.6 ± 2.7	40.4 ± 3.1	38.1 ± 13.3	42.4 ± 2.2	16.1 ± 6.4	20.7 ± 7.6	17.3 ± 7.9	21.2 ± 6.0	28.4 ± 4.5	26.1 ± 4.7
fluct^b^ (Å)	N/A^d^	3.5 ± 0.7	3.4 ± 0.6	3.5 ± 0.8	4.8 ± 1.6	3.8 ± 0.4	4.8 ± 1.4	5.3 ± 1.6	4.6 ± 1.9	5.9 ± 1.3	4.5 ± 1.0	4.7 ± 1.2
RMSD (Å)	N/A^d^	4.2 ± 0.7	4.9 ± 0.7	4.0 ± 0.8	5.2 ± 2.9	5.9 ± 0.4	9.0 ± 1.5	7.4 ± 2.0	7.9 ± 2.2	8.7 ± 2.6	4.9 ± 1.4	5.7 ± 1.4
radgyr^c^ (Å)	12.8	13.6 ± 0.4	14.1 ± 0.3	14.0 ± 0.4	13.0 ± 3.0	14.0 ± 0.3	8.8 ± 1.0	9.9 ± 1.5	9.0 ± 1.6	9.5 ± 16	12.4 ± 1.0	11.6 ± 1.2
volume (MVEE) (Å^3^)	5325	7974 ± 875	8079 ± 841	8311 ± 913	7042 ± 1873	7674 ± 653	6085 ± 787	6551 ± 1032	5853 ± 1140	6902 ± 779	7685 ± 875	7641 ± 890

a End-to-end distance.

b Atomic fluctuation.

c Radius of gyration.

d Atomic fluctuations and root-mean-square deviation (RMSD) are compared to the 1HPN structure; therefore, the values are 0.

**Figure 1:**
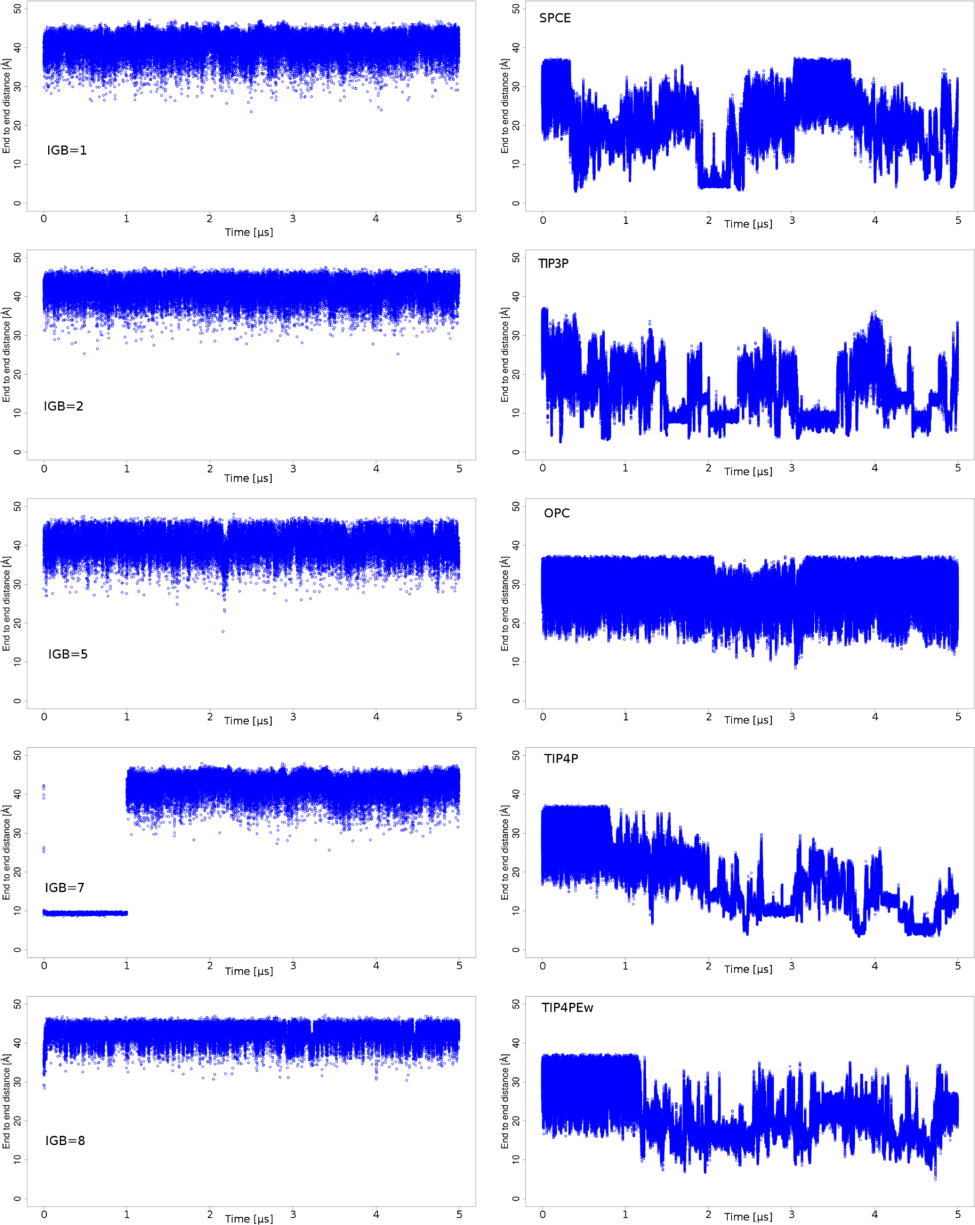

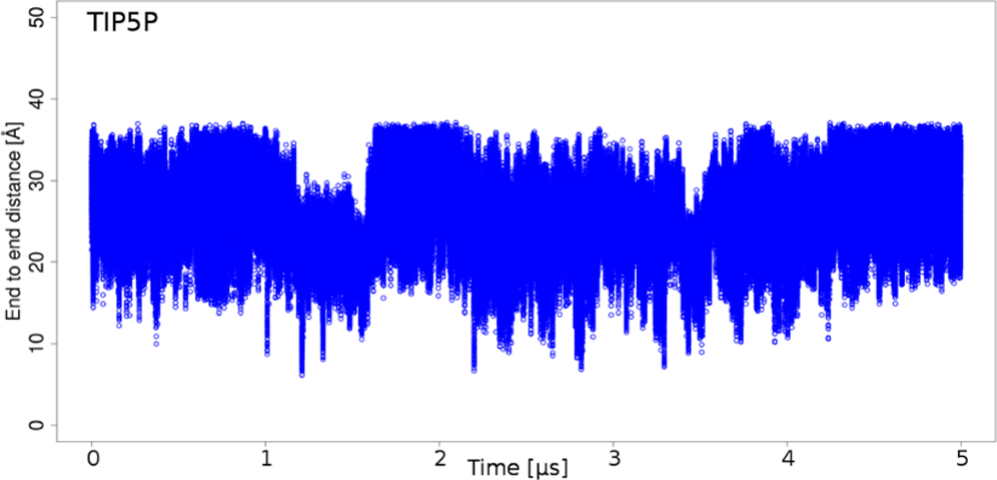
End-to-end distances in the course of 5 μs MD simulations showing differences in HP behavior in different water models. More packed structures can be observed in explicit water models.

Overall end-to-end distances (Figure 1) that were observed during 5 μs are about 2 times bigger and follow unimodal distribution in implicit models. This suggests notably more extended structures in implicit water, especially in IGB = 8. On the contrary, the most curved HP structures were observed in the TIP3P model simulation. Additionally, we observed some drastic and sudden conformational changes in the case of the IGB = 7 model where HP changed the conformation from the extended structure to an “O”-shaped structure (Figure S3) for about 1 μs and then returned back to the extended conformation. This model is known to be not suited for nucleic acids^85^ because Coulomb field approximation (CFA) to define the integral used to calculate effective radii could lead to numerical errors.^70^ We suppose that for HP, which is also highly negatively charged, the reason for the obtained dramatic unnatural structural deformation could be the same.

TIP5P and OPC allowed for the most extended HP structure among the explicit solvent models, which was the closest to the experimental data (41 Å) from the1HPNPDB structure.

Additionally obtained data set was confronted with different experimentally measured EEDs.^86,87^ These EED values are gathered in Table S3. They show that higher curvature was observed only in some of the longer (>dp30) HP molecules. However, when normalized per dp10 (as the length of the HP molecule investigated in this work), the lowest EED was 27.6 Å and the next lowest was 32.3 Å. This is still far away from the values obtained in the TIP3P (16.1 ± 6.4) water model and comparable with the values of HP in the OPC (28.4 ± 4.5 Å) and TIP5P (26.1 ± 4.7 Å) models.

As expected, for the more extended HP structure, the radius of gyration (Table 1 and Figure S4) was higher in GB models, especially in IGB = 5 and 8. The lowest radius of gyration was observed in TIP3P (8.8 Å) water, which is also in agreement with end-to-end distance data. Stand-out explicit water models were OPC (12.4 Å) and TIP5P (11.6 Å) where the radius of gyration of HP was closer to the range of values observed in implicit water models rather than in other explicit ones, which is more in agreement with the findings of Mulloy *et al*.^78^ (12.8 Å) and Khan *et al*. (15.3 Å when normalized for dp10 – 18.3 Å for dp12).^86^

Next, RMSD to the starting point of the MD, which at the same time is the RMSD to the experimental structure since this structure was used as the starting point of the MD runs, was compared. Much higher RMSD (Table 1) was observed in explicit water models than implicit models when compared to the experimental structure (1HPN),^78^ suggesting essentially higher conformational diversity of HP in the former ones. The lowest RMSD was observed in IGB = 5 and IGB = 1 while the highest in TIP3P and TIP4PEw. Again, OPC and TIP5P showed values within the range of those of implicit models rather than within other explicit ones. In those two models, the smallest deviation of the HP structure from the crystal structure was observed among explicit solvent models.

Atomic fluctuations (Table 1) followed a similar trend of the RMSD changes. Again, we observed high values in the case of the TIP4PEw model. However, the difference to the RMSD data here is that in the case of the TIP3P model, values were medium within the explicit models. In the case of implicit models, lowest fluctuations were observed in IGB = 1, 2, and 5.

Additionally, we measured the volume of HP (Table 1 and Figure S5). This volume is defined as a space occupied by the molecule’s atoms and can be used to detect substantial shape/conformational changes of GAG in the MD simulations.^89^ It may be partially dependent on the end-to-end distance and radius of gyration—smaller end-to-end distance and radius of gyration may in some cases mean smaller volume as the molecule in this case is more compact. Details on the way of calculating HP volume are disclosed in the Materials and Methods section. The smallest volume was observed in the case of TIP3P and TIP4P. In the group of explicit water models, HP occupied the largest volume in OPC and TIP5P models and those values were similar to the values observed in the implicit water models. The highest occupied volume was observed in the IGB = 5 water model. However, none of the water models used in this study managed to reproduce experimental findings observed in the1HPNstructure with high accuracy.

**Figure 2:**
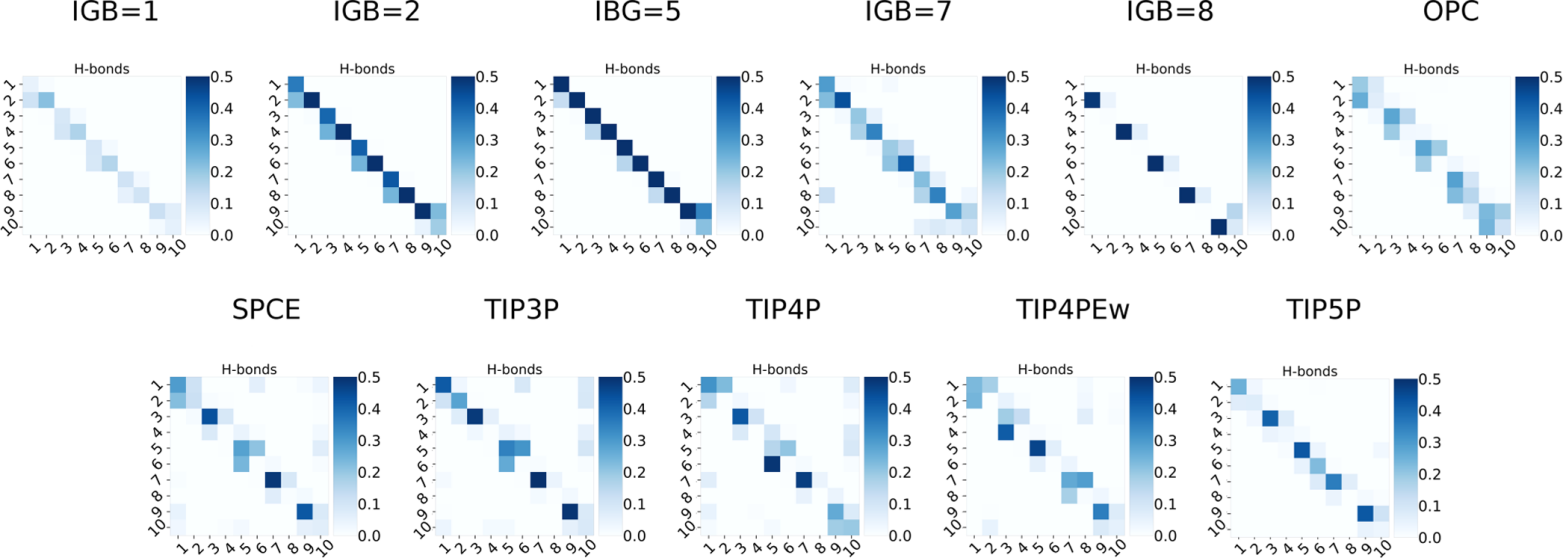
Intramolecular hydrogen bonds established between atoms belonging to the residues in HP (the residue numbers are indicated in both axis labels). More interactions between atoms within the same residue observed in implicit solvent models correspond to the extended conformation. For the explicit solvent models, interactions between different residues can be observed more often suggesting more packed conformations. Dark blue indicates more contacts between residues, light blue shows fewer hydrogen bonds formed, while white space indicates no contacts.

As a next step in the analysis, intramolecular hydrogen bonds were analyzed (Figure 2). In cases when we observed more extended HP structures (GB models and TIP5P), the hydrogen bonds were mostly formed between the atoms belonging to the same residue. However, in the MD runs, when more conformational changes occurred, hydrogen bonds established between atoms in different residues are more often observed. In the case of TIP3P and TIP4P hydrogen bonds between the first and the last GAG residues are formed which indicates a significant amount of the “U”-shaped (Figure S3) HP conformational population during MD simulations, which deviates from the structures observed experimentally. Such conformations are unlikely to occur due to the highly charged nature of the HP molecule, which would rather exclude close proximity of its negatively charged chemical groups in the space. Potentially, such contacts are established in the MD simulation with the TIP3P solvent due to its electrostatic properties allowing for the clustering of counterions that bridge the negatively charged HP groups, which to such an extent was not the case for other, more complex, explicit solvent models (TIP5P and OPC). This phenomenon partially could be explained by similar findings obtained by Pluhařová *et al*.,^91^ where Li2S04 were clustered and closely packed in the explicit solvent (SPC/E model). The observed artifact was caused by strong electrostatic interactions.

**Figure 3:**
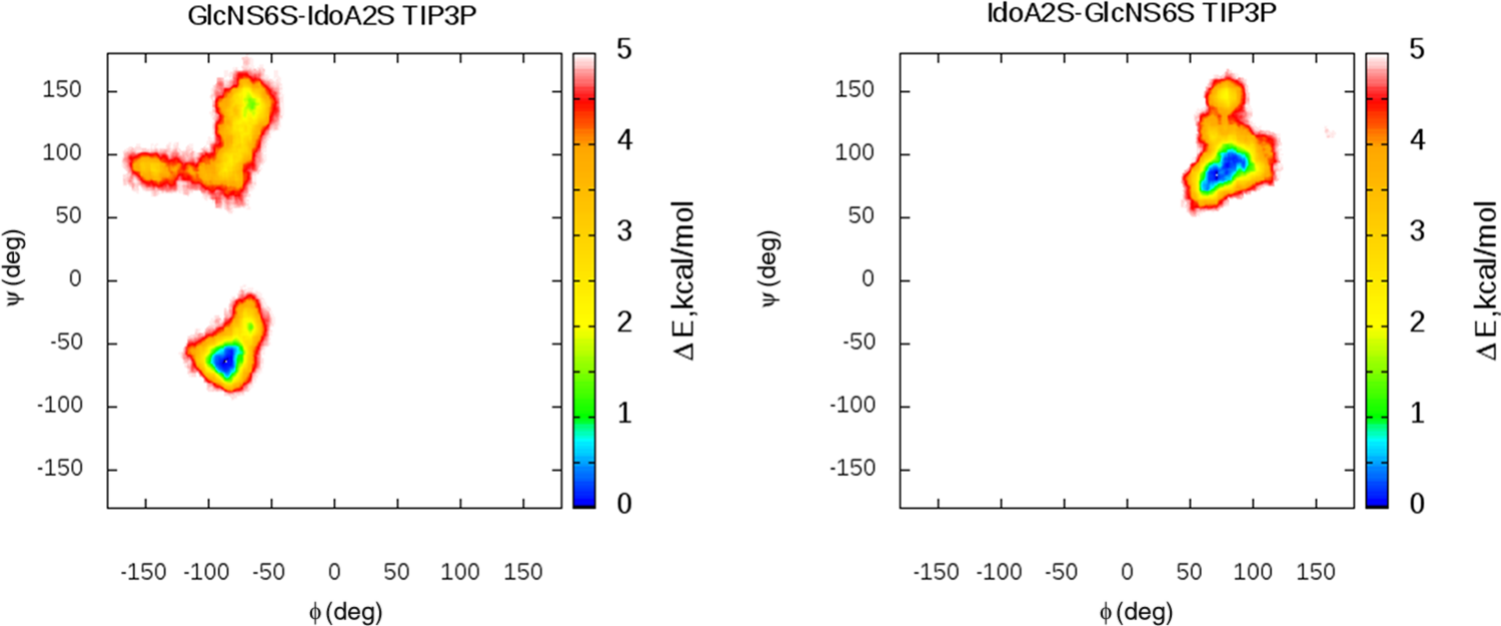
GlcNS6S-IdoA2S and IdoA2S-GlcNS6S glycosidic linkage free energy heatmaps for the TIP3P case. (Glycosidic linkages of the HP molecule in other water models are shown in Figure S4).

Furthermore, φ and ψ dihedral angles for the glycosidic linkages of HP were analyzed (Figures 3 and S6). The heatmaps were obtained by summing up all GlcNS6S-IdoA2S and IdoA2S-GlcNS6S glycosidic linkage-type populations for each of the water models tested. In the case of the widely used TIP3P water, we observe two minima for the GlcN6S-IdoA2S linkage (first φ ∼ −80° and ψ – 50° and second φ ∼ −80° and ψ – 100°) that match experimental/theoretical data^80^ and the computational findings of Bojarski *et al*.^81^ where HP was simulated for 10 μs in complex with a protein. The same applies for the IdoA2S-GlcN6S linkage where we observe a single minimum (φ ∼ 80° and ψ ∼ 100°). For other water models (Figure S6), similar patterns on the heatmaps are observed. However, IGB = 7 data look very concerning as we observe a multiplicity of the various minima that are not present in any of the data known to us (experimental or theoretical). Similar to the work of Holmes *et al*.^92^ performed for heparan sulfate oligosaccharides, we could see that the first GlcN6S-IdoA2S glycosidic linkage minimum (φ ∼ −80° and ψ ∼ −50°) corresponds to the increased curvature of HP, and it is much more pronounced for the explicit solvent where more curved structures are observed. This minimum is barely visible in the heatmaps of the glycosidic linkages in the implicit solvent. The second minimum (φ ∼ −80° and ψ ∼ 100°), which corresponds to the more extended structure, has more favorable energies for the linkages in the implicit solvents. Additionally, for the explicit solvent, this second minimum has more favorable energies in the case of OPC and TIP5P models, and those are the two explicit models where essentially less curvature is observed.

Ring puckering was analyzed to check the effects of a water model that was used on the ring puckering conformations in HP (Tables S4 and S5). In the explicit water environment for the GlcNS6S rings, almost exclusively ^4^C_1_ conformation was observed. The exception were the terminal rings of the GlcNS6S HP unit where some population (up to 13% of the frames) of the ^1^C_4_ conformation was observed. In case of IdoA2S, the ^1^C_4_ conformation was dominant. However, a significant amount of the frames with the ^4^C_1_ ring conformation could be observed (up to 74% in case of the first IdoA2S ring in the TIP3P water model) at the ends of the HP molecule (it is a known issue of the used force field that may cause disruption of the ring conformation at termini of the HP molecule, especially for IdoA derivatives). Very rarely (0–2%), ^2^S_0_ conformation was present. In the implicit water model, all HP rings independently of their type almost exclusively adopted ^4^C_1_ conformation. The exception are IdoA2S residues at the nonreducing end of the HP where 80–100% of the conformations were ^1^C_4_. Another exception is IGB = 1 where 30–100% of the IdoA2S have proper ^1^C_4_ conformation. These data show HP revealing appropriate ring conformations in the explicit solvent (with the exception of the terminal saccharide units of the HP molecule), while in the majority of the implicit water models, they do not reproduce experimental data as all of the IdoA2S ring conformations are disrupted.

End-to-end distance, the radius of gyration, and volume of HP suggest in general more packed structures in the explicit water models. One of the possible reasons for this is the presence of Na^+^ counterions in explicit solvent MD simulations, while these ions are missing in the implicit solvent simulations. In fact, when checked the position of the Na^+^ ions, majority of them were observed in the direct proximity of HP (Figure S3). We suppose that the presence of the ions may favorize the curvature of HP due to Na^+^ interactions with sulfate groups. It was previously reported that ions could stabilize/favor curved structure of other GAGs.^92,93^

To look for the potential changes at a shorter timescale of the MD simulation and to check for the consistency of the calculations, five repeats of the 200 ns MD runs for each of the tested water models were performed. Although overall results were similar (Figures S5 and S6 and Table S1), some differences were observed for the analyzed descriptors in the shorter MD runs. In the case of implicit water models, there were almost no differences between those five 200 ns simulations and 5 μs MD. One exception was IGB = 7 where in some cases we observed unusual behavior of HP that led to the crash of the MD run. The major differences were found for the explicit models. This is due to the fact that the structural features described—stronger curvature, lower volume, and end-to-end distance—were visible after hundreds of ns or even few μs of the MD run. This is the reason why in the shorter simulations in general more extended HP structures were present in comparison to the long MD. In the past, most of the computational studies regarding GAGs were conducted in a shorter timescale. Nowadays, with the availability of stronger computational resources, more advanced longer simulations become common. Before, changes in the long-scale simulations could have been overlooked as they were very rarely investigated. Therefore, it is necessary to put more emphasis on the behavior of the HP in a longer timescale. In this case, TIP3P may not be the best solvent model as after hundreds of nanoseconds, some of the HP properties changed dramatically and did not anymore agree with the experiment.

Additionally, MM/GBSA was applied to investigate the energetic properties of the HP molecule in the surrounding of different water solvent types (Table 2). Most of the HP–water complexes showed energies around −1000 kcal/mol. However, there were some outliers: the highest energies were found in TIP3P (−940 kcal/mol) and TIP4P (−916 kcal/mol) environment, while we observed suspiciously low energies with the IGB = 8 model (−1581 kcal/mol). What is worth mentioning is the fact that obtained energies are in agreement with other observations. In most of the GB models, we observe low variation in the molecular descriptors of HP/small conformational changes during MD simulations, while the corresponding energies do not change much with time—they are the same in shorter 200 ns simulations as in long 5 μs simulation for a respective water model. In the case of several explicit water models where the conformational changes were observed the energies are higher in a 5 μs MD than in 200 ns ones where those changes did not yet occur. This is the case with TIP3P (−940 vs −1006 kcal/mol), SPC/E (−971 vs −1006 kcal/mol), TIP4P (−916 vs −988 kcal/mol), and TIP4PEw (−954 vs −988 kcal/mol). This could suggest that the extended conformation is a major one for both implicit and explicit solvent models. What is important in the case of explicit water models is that energy changes between 200 ns and 5 μs MD in the OPC and TIP5P are the smallest. This again suggests that those two models are able to maintain more proper structural conformation in a longer timescale.

**Table 2:** Energies Obtained from the MM/GBSA Analysis^a^

5 μs simulations											
	IGB = 1	IGB = 2	IGB = 5	IGB = 7	IGB = 8	TIP3P	SPC/E	TIP4P	TIP4PEw	OPC	TIP5P
	–1015 ± 12	–1038 ± 12	–989 ± 12	–1130 ± 141	–1581 ± 12	–940 ± 40	–971 ± 32	–916 ± 53	–954 ± 38	–1007 ± 15	–1005 ± 18

200 ns simulations											
	IGB = 1	IGB = 2	IGB = 5	IGB = 7	IGB = 8	TIP3P	SPC/E	TIP4P	TIP4PEw	OPC	TIP5P
1	–1015 ± 12	–1038 ± 11	–988 ± 12	–1444 ± 43	–1580 ± 12	–1009 ± 15	–999 ± 18	–989 ± 17	–976 ± 31	–1007 ± 14	–1018 ± 13
2	–1014 ± 12	–1038 ± 12	–988 ± 12	–1505 ± 38	–1581 ± 11	–1008 ± 15	–1008 ± 15	–991 ± 19	–972 ± 32	–1009 ± 14	–1016 ± 13
3	–1016 ± 11	–1039 ± 12	–989 ± 11	b	–1581 ± 12	–1008 ± 15	–1008 ± 14	–984 ± 23	–1001 ± 16	–1008 ± 14	–1016 ± 13
4	–1016 ± 12	–1038 ± 12	–988 ± 12	b	–1581 ± 12	–1012 ± 14	–1008 ± 14	–981 ± 26	–1002 ± 16	–1009 ± 14	–1016 ± 13
5	–1016 ± 12	–1038 ± 12	–989 ± 12	–1513 ± 34	–1579 ± 12	–995 ± 20	–1004 ± 15	–996 ± 18	–990 ± 18	–1009 ± 14	–1007 ± 14
average	–1015	–1038	–988	–1487	–1580	–1006	–1006	–988	–988	–1008	–1015

a Values are shown in kcal/mol.

b No data gathered due to crash of the MD simulation.

To sum up, both the local (dihedral angles, puckering) and the global (volume, end-to-end distance, fluctuations, radius of gyration) structural properties of HP in different water models are properly maintained only by TIP5P and OPC models in a longer timescale. Other explicit solvent models allow for proper modeling of the local parameters; however, they failed to maintain global features, which were reflected in the curved/kinked structure of the HP. On the other hand, implicit solvent models tend to preserve properly global parameters of the HP, but they fail to maintain local structural properties.

### Comparison of Solvent Models in the Analysis of Hyaluronic Acid and Chondroitin Sulfate

Additionally, we checked the influence of TIP3P, OPC, and TIP5P water models on MD-derived parameters of hyaluronic acid (HA) and chondroitin sulfate 4 (CS-4). HA and CS-4 have lower charge than heparin; thus, the influence of the solvent model could be expected to be less significant. Considering that the unusual behavior of HP in three-site models could be caused by the fact that electrostatics, which is the driving force in GAGs’ interactions with other molecules, between GAG molecule and ions may not be properly represented. In fact, the differences observed for TIP3P and OPC/TIP5P models in the case of GAGs with lower net charge are not significant (Table 3).

**Table 3:** HA and CS-4 Descriptors Obtained from 5 μs MD Simulations

	HA				CS-4			
	PDB	TIP3P	OPC	TIP5P	PDB	TIP3P	OPC	TIP5P
dist^a^ (Å)	35.9	22.4 ± 5.7	22.5 ± 5.5	24.3 ± 4.9	35.9	23.0 ± 5.2	23.0 ± 5.0	23.2 ± 5.1
fluct^b^ (Å)	N/A^d^	3.5 ± 1.0	3.1 ± 0.8	4.2 ± 1.3	N/A^d^	3.3 ± 1.1	3.1 ± 1.1	3.3 ± 1.1
RMSD (Å)	N/A^d^	4.0 ± 1.4	4.6 ± 0.7	4.7 ± 1.4	N/A^d^	4.0 ± 1.0	3.6 ± 1.0	4.1 ± 1.3
radgyr^c^ (Å)	11.9	14.0 ± 1.0	14.1 ± 0.7	12.8 ± 1.1	11.9	13.8 ± 0.7	13.8 ± 0.7	13.4 ± 0.8
volume (MVEE) (Å^3^)	7426	7331 ± 1288	7353 ± 1158	7769 ± 1145	7375	8696 ± 1192	8620 ± 1211	9096 ± 1178

a End-to-end distance.

b Atomic fluctuation.

c Radius of gyration.

d Atomic fluctuations and RMSD are compared between the experimental structures of HA and CS-4 (PDB IDs: 1HYA and 1C4S, respectively) and the analyzed structures, therefore, the values are 0.

### Future Perspective for the Use of Water Models in GAG Studies

Although TIP5P and OPC performed the best in this study when analyzing different HP properties, we urge to treat these results with caution. This study only investigated free HP in the solution and not in the presence of proteins, which potentially could affect the drawn conclusions. In our previous studies involving MD of protein–GAG complexes, we did not observe any unusual behavior of the GAG molecules in the TIP3P solvent. Moreover, TIP5P and OPC seem to only provide improvement in the case of highly charged GAG molecules. In the case of GAGs with a lower negative charge, no significant differences were observed in their behavior in TIP3P and TIP5P or OPC. Therefore, more testing studies in this regard are required. Additionally, we do not find any strong thermodynamic rationale for justification of TIP5P/OPC improvement on the HP structural behavior in the available literature. It also has to be noted that GLYCAM06 was initially tested with TIP3P as a primary water model, which theoretically should provide optimal or at least satisfactory results in the simulations. Taking all of this into account, we recommend careful consideration of used water models in the GAG computational studies. We are certain that further studies are required to fully unravel the impact of the water models on the behavior and structural properties of the GAG molecules.

## Conclusions

The observed values of MD-based molecular descriptors for HP differed significantly between the groups of implicit and explicit water models used in the simulations. Some interesting features were observed, e.g., HP in OPC and TIP5P models behave similarly to the implicit water models maintaining global properties in agreement with the experimental data. The IGB = 7 model caused sometimes abnormal behavior of HP, and improper glycosidic linkage populations were observed. In general, the implicit solvent models do not even qualitatively agree with the experiment in terms of reproducing the HP ring puckering. Therefore, we do not suggest using implicit models for simulating GAGs unless the restraints are applied to maintain the rings in particular conformations reproducing specific experimental data. The most distinguishable model among the explicit ones was the TIP3P water model, one of the most commonly used models in biomolecular systems in general, in which HP displayed completely different properties than in the implicit models. In the case of TIP3P (and other explicit models, but to lesser extent) we observed characteristic curvature of HP as it was often “U”-shaped. It might be due to the fact that in the case of the explicit water model, there are counterions and thus heavily charged HP willingly interacts with those ions and trap them inside. The reason why we observe this notably less in the case of OPC and TIP5P models might be the fact that those models are much more complex and thus they can provide more appropriate charge distribution leading to a more physically relevant electrostatic description. It is of immense importance as TIP3P is a widely used water model in the studies of GAGs, but here, evidences were presented that in a longer (microsecond range) timescale, TIP3P may fail to maintain proper structural conformational ensemble of the HP molecule. When using all explicit solvent models, HP rings kept the proper conformations (ring puckering) in agreement with the experimental data, while in majority of the implicit models, IdoA2S rings revealed improper conformations. In our previous research, we did not observe any unusual behavior of HP (or other GAG molecules) as extensive curvature, packed conformation, wrong glycosidic dihedral or pucker angles, etc. However, in all of our previous studies (and vast majority of the studies that are conducted by other groups), GAG molecules are simulated in complex with the proteins, or the MD simulations are significantly shorter than 5 μs. In the former case, HP interacts with the charged protein and is unable to freely cluster the ions, which could potentially induce the bend. This could prevent the unusual behavior of the GAG in which case ions are localized inside the curved GAG structure. Therefore, we strongly believe that the effects of water models on HP properties need to be extensively studied together in the presence of protein molecules in a long MD simulation. Similarly, in the work of Neamtu *et al*.,^94^ four- and five-site water models performed better than TIP3P in reproducing several structural features of chondroitin sulfate. However, in this study, the first layer of hydration was found to be better represented by the TIP3P model, and it was also found that TIP3P favors the direct intramolecular hydrogen bonding of chondroitin sulfate while the four-site and five-site models disfavor it. Additionally, when using the TIP5P model, a better-defined long-range hydration layer order around the hydroxyl groups of the sugar ring was observed compared with the other studied models. Within the nowadays standards, GAG molecules are almost exclusively simulated in the TIP3P model and it still remains necessary to confirm if it is the appropriate model for the GAG–protein simulations or to select a better one. In particular, this question is justified by considering the fact that in 5 μs simulations, TIP3P failed to maintain proper global structural features of the HP, and the HP conformations deviated essentially from the one observed in the experiment. The only two solvent models that allowed for reproduction of both proper local and global structural features of the HP in the MD simulation were TIP5P and OPC. What also needs to be emphasized is the fact that the preliminary investigation of GAGs with lower charge than by HP did not reveal any significant differences in the GAG properties depending on the use of different water models (either TIP3P or TIP5P/OPC). Considering both results for the HP study and less charged GAGs, we would like to spark a discussion regarding the use of water models in theoretical GAG-related studies and whether any changes in the established approaches are required. Additionally, we would like to encourage other computational groups interested in GAGs to contribute to this potentially very important topic, especially considering this type of analysis of multiple GAG–protein complexes would be highly beneficial and could shed more light on this highly relevant subject in the GAG theoretical research.

## Data Availability

All of the data (MD simulations) were obtained using AMBER suite (Amber20 and AmberTools). The data were then analyzed using R (statistics and plots), VMD (visualization of the obtained trajectories), and GIMP (figure preparation). The data used to generate MD trajectories are accessible in a publicly available repository Zenodo under the link https://doi.org/10.5281/zenodo.7260781. All software except the AMBER suite is free of charge. AMBER software can be obtained from http://ambermd.org/GetAmber.php. R can be downloaded from https://www.r-project.org/. GIMP can be downloaded from https://www.gimp.org/. VMD can be downloaded from http://www.ks.uiuc.edu/Research/vmd/.

## Supporting Information Available

Graphical representation of water model types used in this study together with their detailed description; visualization of the atoms chosen for the calculations of the dihedral angles and glycosidic linkages; visualization of the HP structures obtained during MD simulations; auxiliary graphs representing different parameters analyzed in this work; auxiliary tables gathering more information regarding investigated parameters of GAG molecules; and comparison of experimentally reported end-to-end distances (PDF)
