# Supplementary material for: Solvent Model Benchmark for Molecular Dynamics of Glycosaminoglycans
Source: J Chem Inf Model. 2023 Mar 29;63(7):2147–57. doi: 10.1021/acs.jcim.2c01472 (PMC10091405; doi:10.1021/acs.jcim.2c01472)
Supplement: Supplementary file 1 — ci2c01472_si_001.pdf [file ci2c01472_si_001.pdf]

Supporting Information

Solvent models benchmark for molecular dynamics of glycosaminoglycans

Mateusz Marcisz[1,2*], *Sergey A. Samsonov*[1*]

[1]Faculty of Chemistry, University of Gdańsk, ul. Wita Stwosza 63, 80-308 Gdańsk, Poland.

[2]Intercollegiate Faculty of Biotechnology of UG and MUG, ul. Abrahama 58, 80-307 Gdańsk, Poland.

**Corresponding authors:* Mateusz Marcisz (mateusz.marcisz@phdstud.ug.edu.pl) and Sergey A. Samsonov (sergey.samsonov@ug.edu.pl)

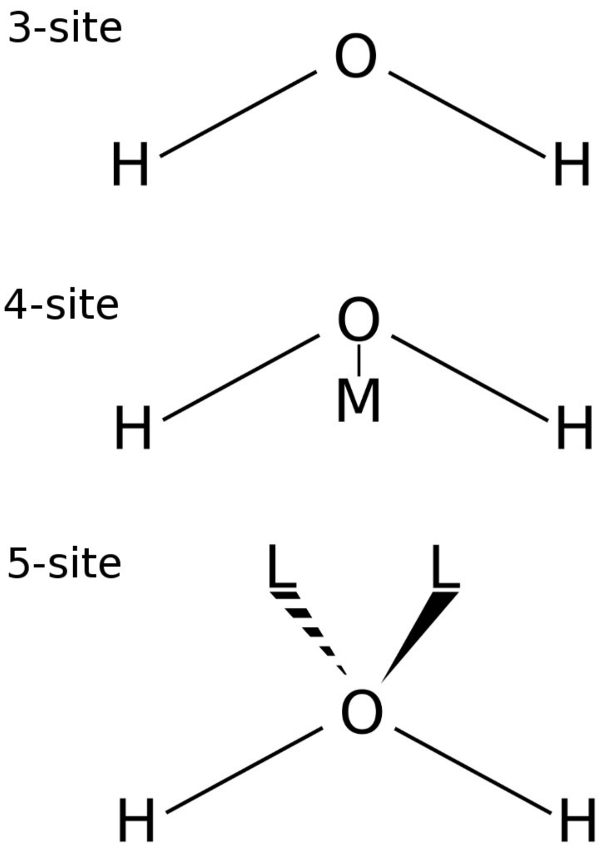

Figure S1. Representation of water model types in this study. 3-site models used: TIP3P, SPC/E. 4-site models used: TIP4P, TIP4PEw, OPC. 5site model used: TIP5P. “M” in the 4-site model represents dummy atom with the negative charge (-1.04, -1.04844 and -1.3582 for the TIP4P, TIP4PEw and OPC respectively). The “L” in the 5-site model represents the lone pairs of the oxygen atom. L has the charge of -0.241 in the TIP5P model. The HOH angle in the TIP3P, TIP4P, TIP4PEw andTIP5Pis 104.52 deg. In case of the SPC/E HOH angle is 109.47 deg and for the OPC HOH angle is 103.6 deg. Distance between O and H is 0.9572 Å for the TIP3P, TIP4P, TIP4PEw and TIP5P, 0.8724 Å for the OPC and 1 Å for the SPC/E. In case of 4-site models distance between O and the dummy atom M is 0.15 Å for the TIP4P, 0.125 Å for the TIP4PEw and 0.1594 Å for the OPC. In case of 5-site model the distance between O and lone pairs is 0.7 Å for the TIP5P and the angle is 109.47 deg.

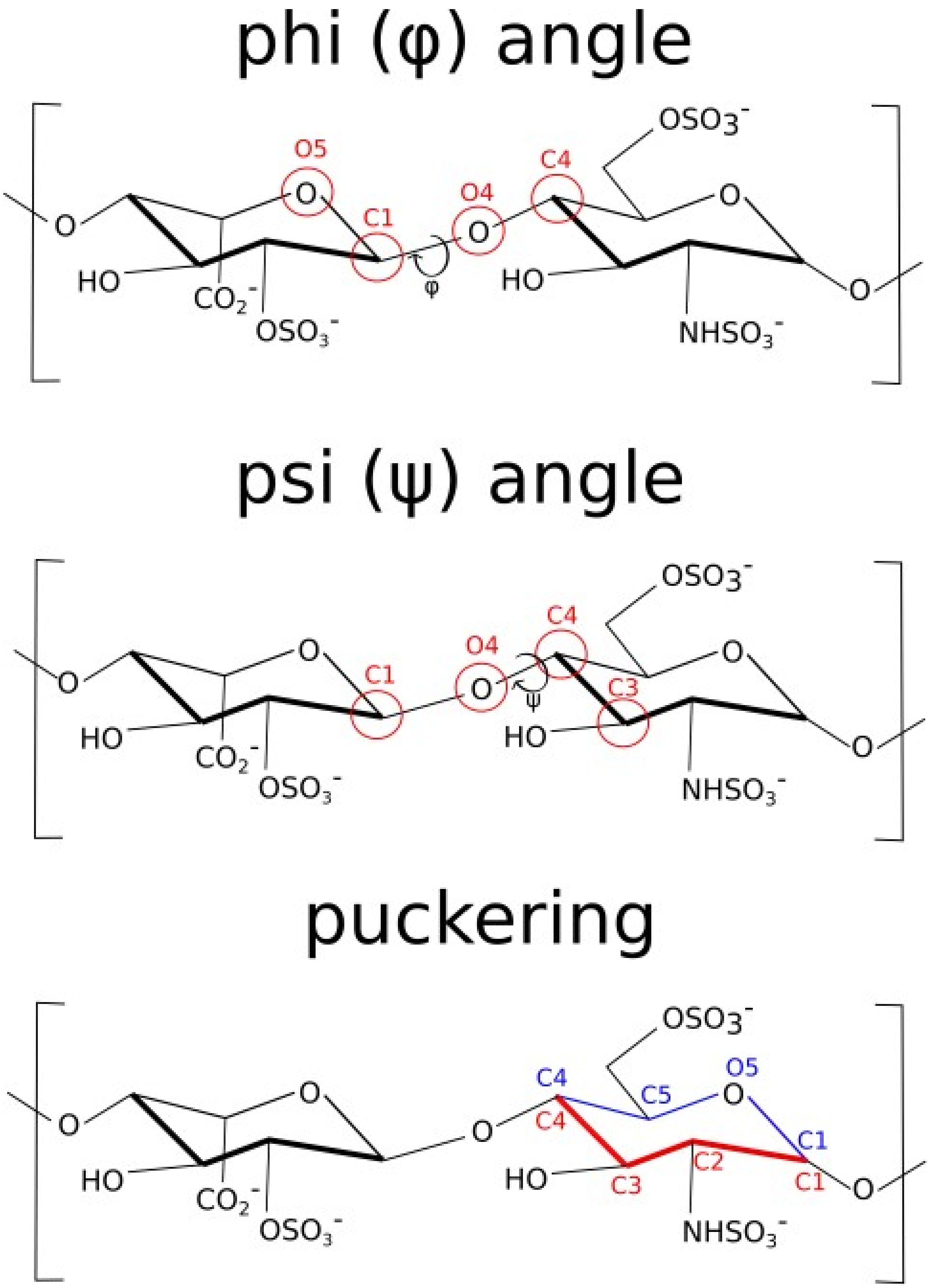

Figure S2. Visualization of the atoms chosen for the calculations of the dihedral angles. For the glycosidic linkages (psi and phi angles) atoms are highlighted in red circles and colored in red. For the puckering for the one angle atoms are colored in blue and for the second angle in red. Numbers of atoms correspond to the numbering used in AMBER.

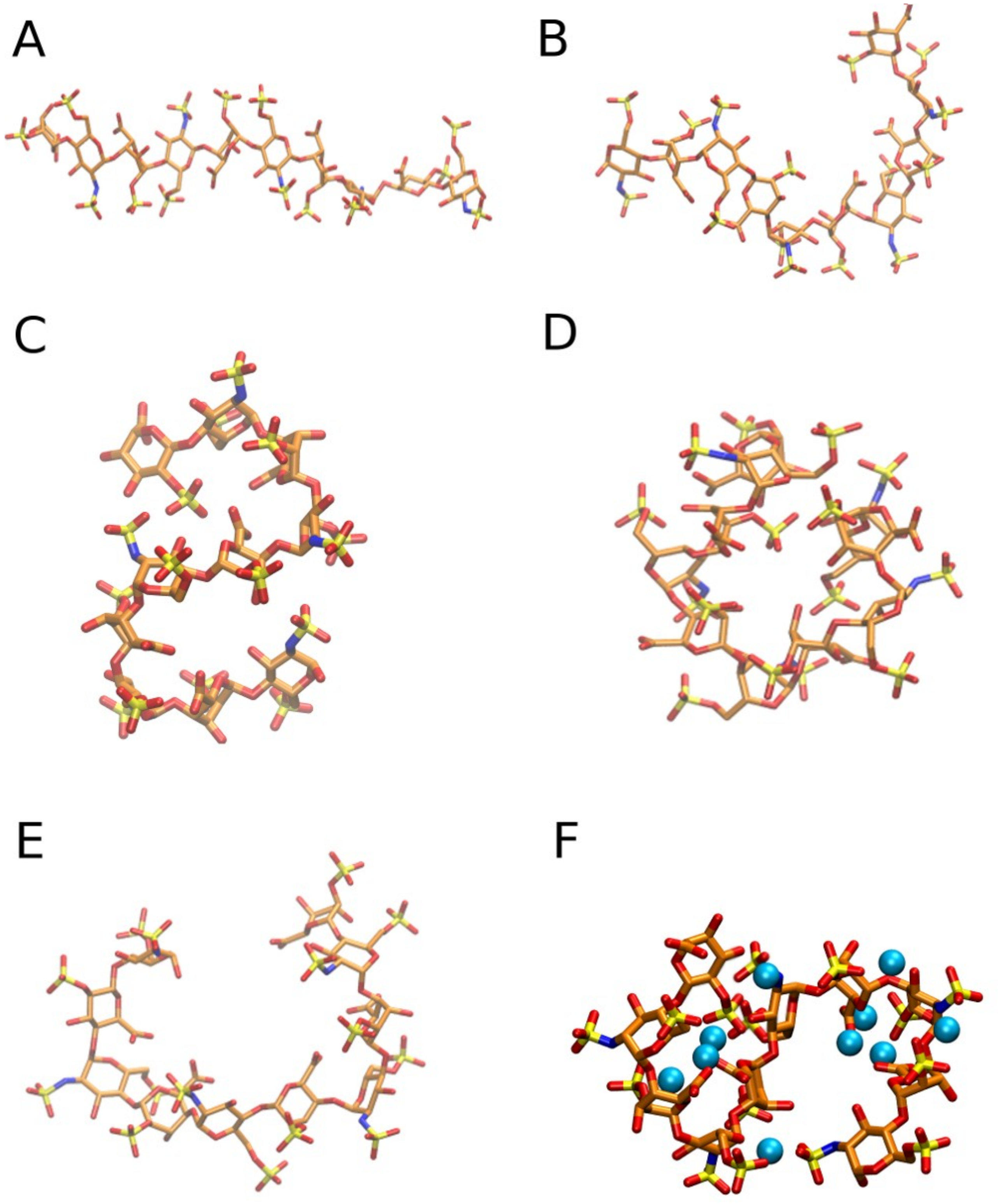

Figure S3. Visualization of the HP structures (licorice) obtained during MD simulations. A: extended structure. B: Semi-extended structure. C: “S” structure. D. “O” structure. E: “U” structure. F: Bended structure with ions (clue spheres).

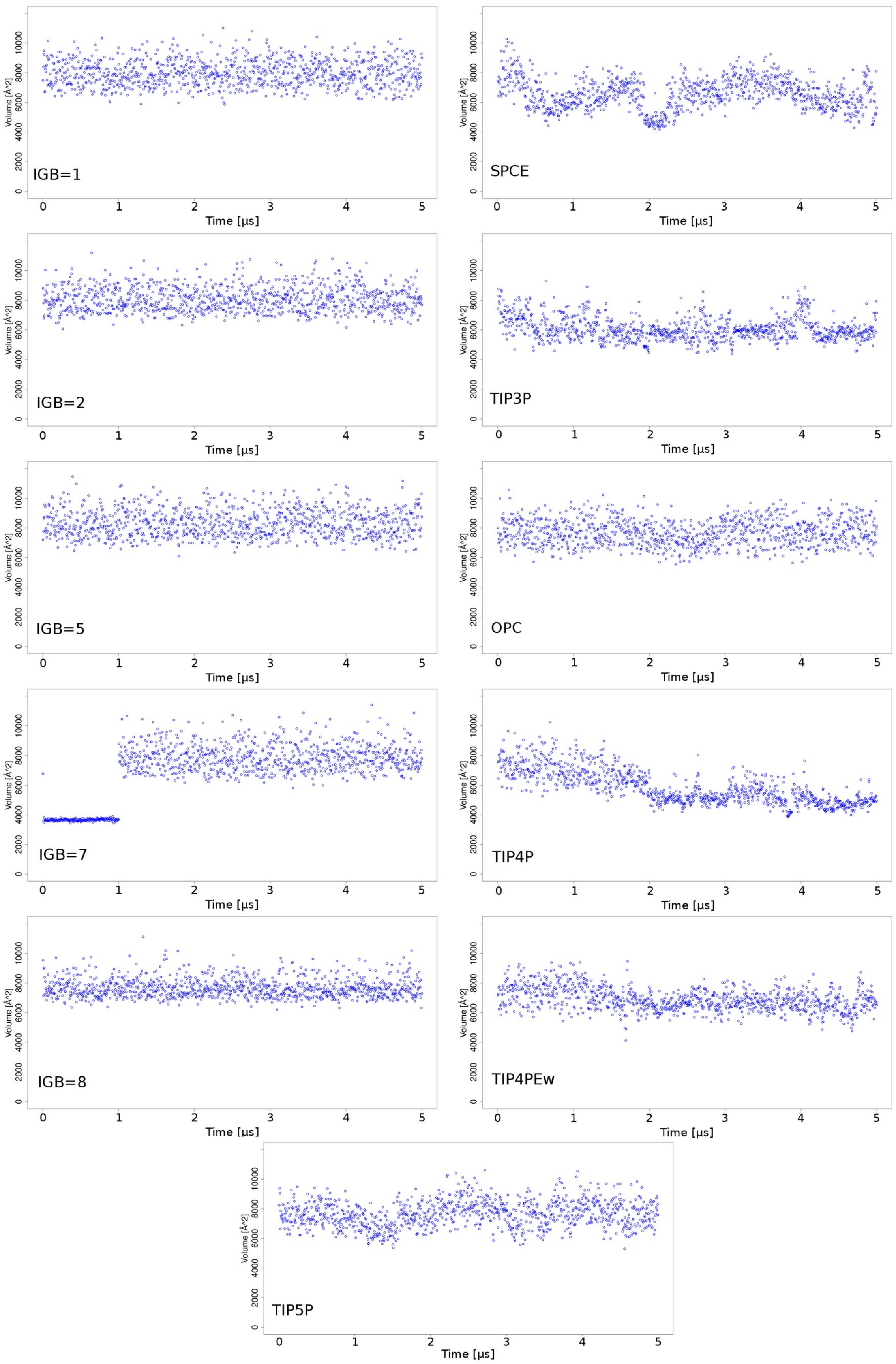

Figure S4. Volume of the HP in the MD simualtions with different water models.

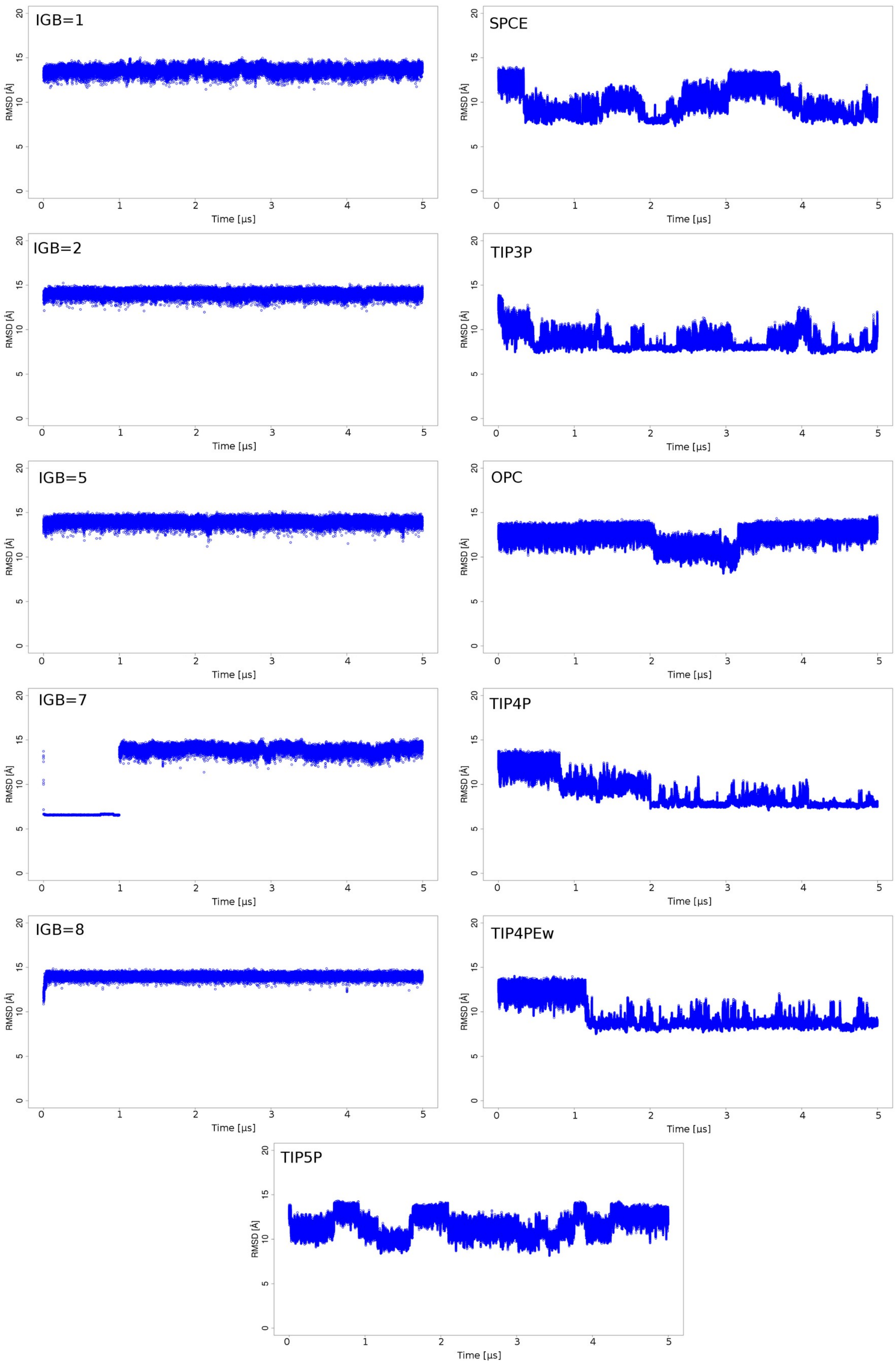

Figure S5. Radius of gyration of the HP in the MD simualtions with different water models.

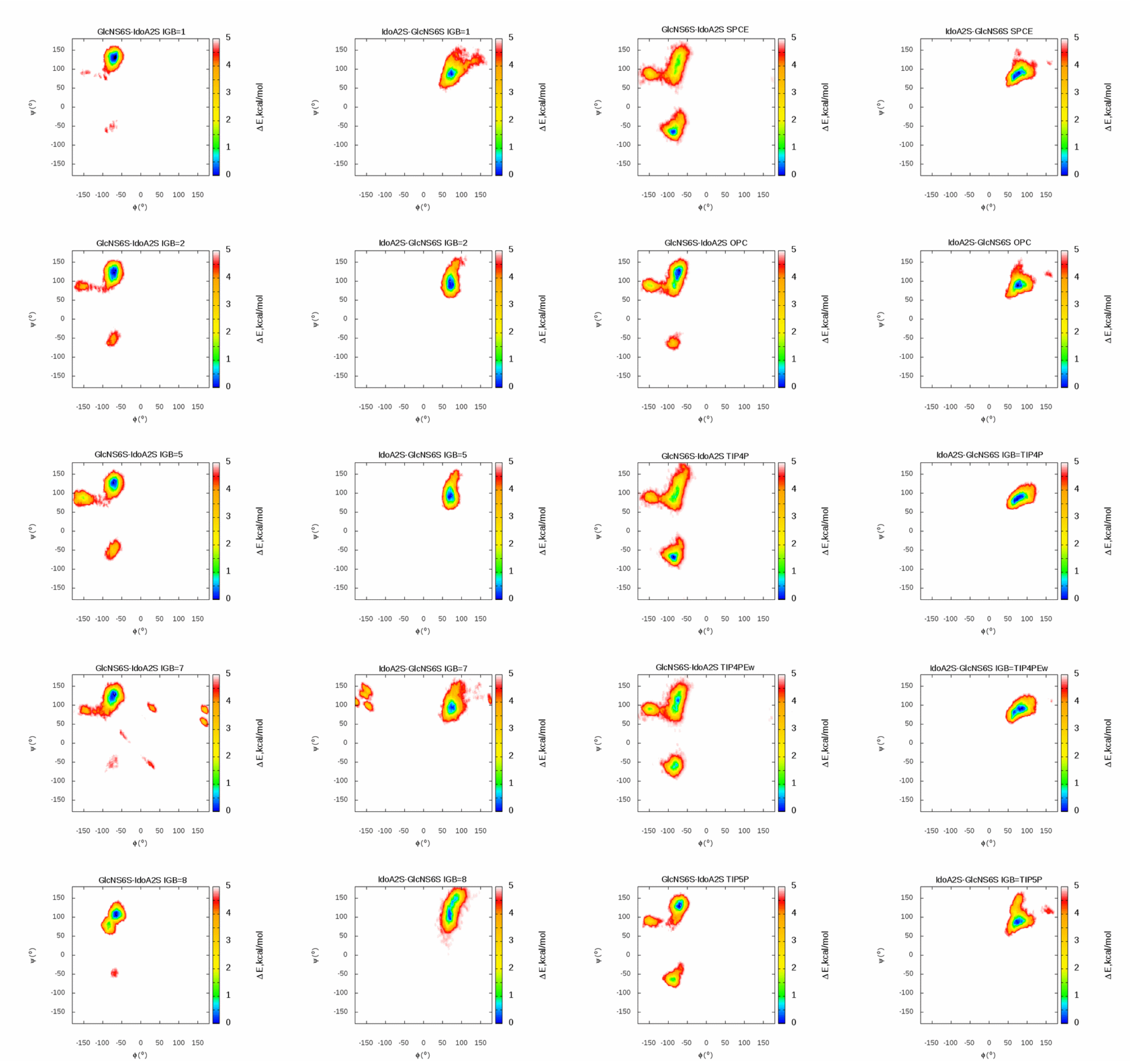

Figure S6. GlcNS6S-IdoA2S and IdoA2S- GlcNS6S glycosidic linkage energy heatmaps in the MD simualtions with different water models.

Table S1. Descriptors obtained during 5 x 200 ns simulations describing HP properties. dist = End to end distance. fluct = Atomic fluctuation. radgyr = Radius of gyration.

		5 x 200 ns				
		IGB=1	IGB=2	IGB=5	IGB=7	IGB=8
dist	1.0	40.3	40.9	40.7	4.4	40.9
	2.0	41.1	41.9	39.8	7.6	42.3
	3.0	40.1	40.8	39.4	error	41.9
	4.0	40.1	41.1	40.3	error	41.2
	5.0	40.2	41.5	40.2	13.0	40.8
	mean	40.4	41.2	40.1	8.4	41.4
fluct	1.0	3.9	3.1	3.5	error	3.6
	2.0	3.6	3.2	3.4	2.0	3.6
	3.0	3.5	3.3	3.6	error	2.8
	4.0	3.5	3.2	3.4	error	3.6
	5.0	3.5	3.2	3.7	0.6	3.8
	mean	3.6	3.2	3.5	1.3	3.5
rmsd	1.0	5.8	3.9	4.4	12.0	5.7
	2.0	4.3	4.0	4.0	10.4	5.9
	3.0	3.9	4.0	4.1	error	4.7
	4.0	4.0	4.0	3.7	error	7.0
	5.0	3.9	4.2	4.6	8.1	5.8
	mean	4.4	4.0	4.2	10.2	5.8
radgyr	1.0	13.5	13.9	13.9	6.6	13.7
	2.0	13.8	14.0	13.9	6.9	14.0
	3.0	13.4	13.9	13.7	error	13.9
	4.0	13.4	13.9	13.8	error	13.9
	5.0	13.4	14.1	13.9	7.2	13.7
	mean	13.5	14.0	13.9	6.9	13.8
volume	1.0	7879.3	8259.4	8257.3	4223.4	8012.6
	2.0	7966.3	7956.1	8208.7	4345.9	7795.0
	3.0	7864.1	7999.6	8352.3	error	7736.4
	4.0	7777.5	8040.0	8016.8	error	7745.5
	5.0	7733.4	8329.8	8199.3	3360.2	8054.1
	mean	7844.1	8117.0	8206.9	3976.5	7868.7

Table S2. Descriptors obtained during 5 x 200 ns simulations describing HP properties. dist = End to end distance. fluct = Atomic fluctuation. radgyr = Radius of gyration. errror = simulations not finished due to the crush

5 x 200 ns						
	TIP3P	SPC/E	TIP4P	tip4pEw	OPC	TIP5P
dist	30.0	26.0	30.1	26.6	29.3	29.6
	29.6	29.4	29.3	21.0	29.4	29.1
	29.3	29.4	28.6	29.6	29.6	28.4
	29.5	29.5	28.6	29.3	30.1	28.4
	25.6	30.2	29.7	26.5	29.9	26.6
	28.8	28.9	29.2	26.6	29.7	28.4
fluct	4.0	4.1	4.8	4.1	3.6	4.0
	4.2	3.7	5.3	4.7	3.6	3.6
	3.7	3.8	5.7	3.7	3.7	5.3
	3.8	4.0	5.6	3.9	3.8	4.0
	4.5	3.6	3.7	4.3	3.7	3.9
	4.1	3.9	5.0	4.2	3.7	4.2
rmsd	4.1	5.9	4.4	6.7	3.9	4.1
	4.5	4.0	4.5	8.5	4.1	3.8
	4.3	4.3	5.3	4.2	4.2	4.1
	4.4	4.2	4.8	4.0	4.2	4.7
	7.0	4.2	4.1	6.0	4.1	6.6
	4.8	4.5	4.6	5.9	4.1	4.7
radgyr	12.5	11.2	12.1	10.3	12.5	12.7
	12.5	12.4	12.1	9.8	12.6	12.8
	12.5	12.4	11.4	12.3	12.5	12.8
	12.5	12.4	11.8	12.3	12.6	12.6
	10.9	12.2	12.2	11.2	12.5	11.1
	12.2	12.1	11.9	11.2	12.6	12.4
volume	7603.3	7106.0	7397.4	7223.6	7481.6	7575.4
	7562.1	7509.5	7315.6	7014.5	7605.6	7494.2
	7741.5	7655.2	7413.5	7371.5	7668.2	7686.1
	7609.0	7639.6	7137.3	7434.7	7656.9	7769.4
	7448.1	7374.9	7384.6	7284.7	7639.8	8156.4
	7592.8	7457.0	7329.7	7265.8	7610.4	7736.3

Table S3. Different experimentally measured EEDs (End to end distances) and normalized per dp10 EEDs.

	EED Khan *et al* [Å]	EED Khan *et al* normalized per10 [Å]	EED Pavlov *et al* [Å]	EED Pavlov *et al* normalized for dp10 [Å]
dp12	60	50	-	-
dp18	74	41.1	-	-
dp24	94	39.2	-	-
dp30	103	34.3	-	-
dp32	-	-	125	39.1
dp36	123	34.2	-	-
dp48	-	-	155	32.3
dp68	-	-	188	27.6

Table S4. Ring conformation populations (%) for HP in the MD simulations with different explicit water models.

				Explicit water models					
TIP3P	$^{1}C_{4}$	$^{4}C_{1}$	$^{2}S_{0}$	$^{1}S_{3}$	SPCE	$^{1}C_{4}$	$^{4}C_{1}$	$^{2}S_{0}$	$^{1}S_{3}$
ROH-1	12.72	86.32	0.04	0.12	ROH-1	6.98	92.18	0.02	0.04
2	24.90	73.96	0.78	0.02	2	74.44	24.28	0.72	0.02
3	0.00	99.88	0.00	0.00	3	0.00	99.72	0.02	0.00
4	99.22	0.00	0.48	0.00	4	99.28	0.00	0.52	0.02
5	0.00	99.64	0.00	0.04	5	0.00	99.60	0.00	0.00
6	98.52	0.00	1.34	0.08	6	99.00	0.00	0.80	0.04
7	0.00	99.82	0.00	0.00	7	0.00	99.68	0.00	0.00
8	99.40	0.00	0.52	0.00	8	98.46	0.00	1.20	0.10
9	0.00	99.80	0.00	0.00	9	0.00	99.80	0.00	0.00
10	91.88	7.64	0.18	0.02	10	99.74	0.00	0.0	0.00
TIP4P	$^{1}C_{4}$	$^{4}C_{1}$	$^{2}S_{0}$	$^{1}S_{3}$	TIP4PEw	$^{1}C_{4}$	$^{4}C_{1}$	$^{2}S_{0}$	$^{1}S_{3}$
ROH-1	0.00	99.44	0.04	0.04	ROH-1	3.42	95.82	0.00	0.02
2	98.84	0.00	0.92	0.06	2	99.04	0.00	0.08	0.3
3	0.00	99.78	0.00	0.00	3	0.00	99.72	0.00	0.00
4	99.32	0.00	0.46	0.02	4	97.86	0.00	1.80	0.08
5	0.00	99.88	0.00	0.00	5	0.00	99.66	0.00	0.00
6	99.04	0.00	0.84	0.04	6	99.32	0.00	0.62	0.02
7	0.00	99.92	0.00	0.00	7	0.00	99.76	0.00	0.00
8	99.76	0.00	0.14	0.02	8	99.42	0.00	0.42	0.00
9	0.00	98.80	0.00	0.00	9	0.00	99.80	0.00	0.00
10	54.24	44.02	0.98	0.10	10	99.66	0.00	0.06	0.04
OPC	$^{1}C_{4}$	$^{4}C_{1}$	$^{2}S_{0}$	$^{1}S_{3}$	TIP5P	$^{1}C_{4}$	$^{4}C_{1}$	$^{2}S_{0}$	$^{1}S_{3}$
ROH-1	0.00	99.44	0.00	0.02	ROH-1	10.44	88.62	0.02	0.08
2	83.08	15.74	0.98	0.10	2	79.82	19.14	0.46	0.02
3	0.00	99.74	0.00	0.00	3	0.00	99.78	0.00	0.00
4	96.54	2.20	1.08	0.02	4	95.22	3.56	1.06	0.02
5	0.00	99.74	0.00	0.00	5	0.00	99.76	0.00	0.00
6	99.04	0.00	0.80	0.02	6	21.72	77.10	0.10	0.00
7	0.00	99.94	0.00	0.00	7	0.00	99.78	0.00	0.00
8	48.16	50.14	1.26	0.02	8	99.50	0.00	0.38	0.00
9	0.00	99.76	0.00	0.00	9	0.00	99.90	0.00	0.00
10	92.22	6.94	0.54	0.04	10	99.54	0.00	0.18	0.00

Table S5. Ring conformation populations for HP in the MD simulations with different implicit water models.

Implicit water models

IGB=1	$^{1}C_{4}$	$^{4}C_{1}$	$^{2}S_{0}$	$^{1}S_{3}$	IGB=2	$^{1}C_{4}$	$^{4}C_{1}$	$^{2}S_{0}$	$^{1}S_{3}$
ROH-1	6.54	92.24	0.08	0.06	ROH-1	1.06	97.08	0.10	0.50
2	29.54	63.22	5.84	0.16	2	1.42	93.54	3.82	0.24
3	1.08	98.10	0.02	0.06	3	0.06	99.32	0.00	0.00
4	45.18	43.54	10.34	0.20	4	1.82	89.78	7.04	0.10
5	4.98	94.12	0.02	0.04	5	0.00	99.38	0.04	0.02
6	51.56	37.90	9.48	0.18	6	2.08	89.22	7.34	0.14
7	1.86	97.16	0.02	0.06	7	0.00	99.08	0.00	0.02
8	67.60	22.50	9.38	0.20	8	4.26	82.58	11.88	0.24
9	0.06	98.98	0.00	0.02	9	0.00	99.32	0.02	0.04
10	99.50	0.10	0.16	0.00	10	95.14	2.96	1.56	0.14
IGB=5	$^{1}C_{4}$	$^{4}C_{1}$	$^{2}S_{0}$	$^{1}S_{3}$	IGB=7	$^{1}C_{4}$	$^{4}C_{1}$	$^{2}S_{0}$	$^{1}S_{3}$
ROH-1	1.08	96.96	0.32	0.68	ROH-1	1.56	77.06	0.08	0.22
2	0.40	96.42	1.98	0.04	2	12.32	67.02	1.72	0.38
3	0.00	99.46	0.02	0.02	3	0.02	99.44	0.02	0.02
4	0.88	88.94	8.84	0.16	4	45.84	48.88	4.50	0.22
5	0.00	99.36	0.00	0.04	5	0.28	82.40	0.00	13.06
6	1.08	89.60	8.28	0.16	6	23.36	62.44	1.98	0.04
7	0.00	99.16	0.00	0.00	7	0.08	96.48	0.02	0.26
8	3.04	77.14	18.64	0.42	8	50.24	45.68	3.48	0.06
9	0.32	99.02	0.02	0.02	9	0.08	99.34	0.00	0.00
10	96.18	0.76	2.54	0.28	10	79.82	5.10	0.50	0.70
IGB=8	$^{1}C_{4}$	$^{4}C_{1}$	$^{2}S_{0}$	$^{1}S_{3}$					
ROH-1	0.60	98.54	0.02	0.02					
2	0.42	99.30	0.06	0.00					
3	0.00	99.40	0.00	0.00					
4	0.20	99.30	0.06	0.02					
5	0.18	99.04	0.00	0.00					
6	0.16	99.40	0.04	0.00					
7	0.00	99.26	0.00	0.00					
8	0.66	98.90	0.06	0.00					
9	0.00	99.28	0.00	0.00					
10	99.34	0.40	0.02	0.00					
